# A Case of Pediatric Sternal Fracture Diagnosed by POCUS

**DOI:** 10.24908/pocus.v8i1.15824

**Published:** 2023-04-26

**Authors:** Takaaki Mori, Sung Shin Teng

**Affiliations:** 1 Department of Emergency Medicine, KK Women's and Children's Hospital Singapore

**Keywords:** point-of-care ultrasound, sternal fracture, children

## Abstract

A previously healthy, 4-year-old boy visited our emergency department due to chest pain after a fall from a skate scooter. Physical examination revealed tenderness over the sternal body. Point of care ultrasound (POCUS) of the sternum demonstrated a discontinuation of a hyperechoic structure of the sternal cortex, suggesting a sternal fracture. POCUS did not detect intraperitoneal fluid, pericardiac effusion, or pneumothorax. Plain radiograph confirmed the diagnosis of isolated sternal fracture and the patient was discharged with conservative treatment. POCUS was useful not only in diagnosing a sternal fracture but also to rule out concurrent injuries.

## Introduction

Sternal fractures in children are uncommon because of the marked chest compliance 1. The most common mechanism of pediatric sternal fractures is motor vehicle accident, and the widespread use of seat belt has contributed to the increase in this injury 2. Sternal fractures sometimes associate with comorbidities such as pneumothorax, pulmonary contusion, cardiac injury, and extremity fractures, alarming physicians to perform extensive examinations and investigations to exclude these complications 3, 4. However, isolated sternal fractures can be treated conservatively 5. Radiography (anteroposterior and lateral view) is usually used for diagnosing sternal fractures, but proper interpretation of the radiography is sometimes difficult, and it is often challenging to obtain lateral radiography in traumatized patients 6. Computed tomography (CT) or magnetic resonance imaging (MRI) are accurate diagnostic modalities for diagnosing sternal fractures but they are undesirable in pediatric patients because they involve procedural sedation or radiation exposure 7. Point of care ultrasound (POCUS) is an alternative modality for diagnosing fractures 8, but the reports of its use for diagnosing sternal fractures in the pediatric emergency care setting are scarce. We herein report a pediatric case of sternal fracture detected by pediatric emergency physician-performed POCUS. 

## Case Report 

A previously healthy, 4-year-old boy visited our emergency department due to chest pain. Chest injury was sustained from hitting the handle bar while falling off a skate scooter. He denied any accompanying symptoms such as palpitation, syncope, or vomiting. He was alert and his vital signs were appropriate for his age. Physical examination revealed tenderness over the sternal body, but no tenderness over the ribs or abdomen. His lung auscultation was clear with bilateral equal air entry, and his heart rhythm was regular without murmurs. 

**Figure 1:**
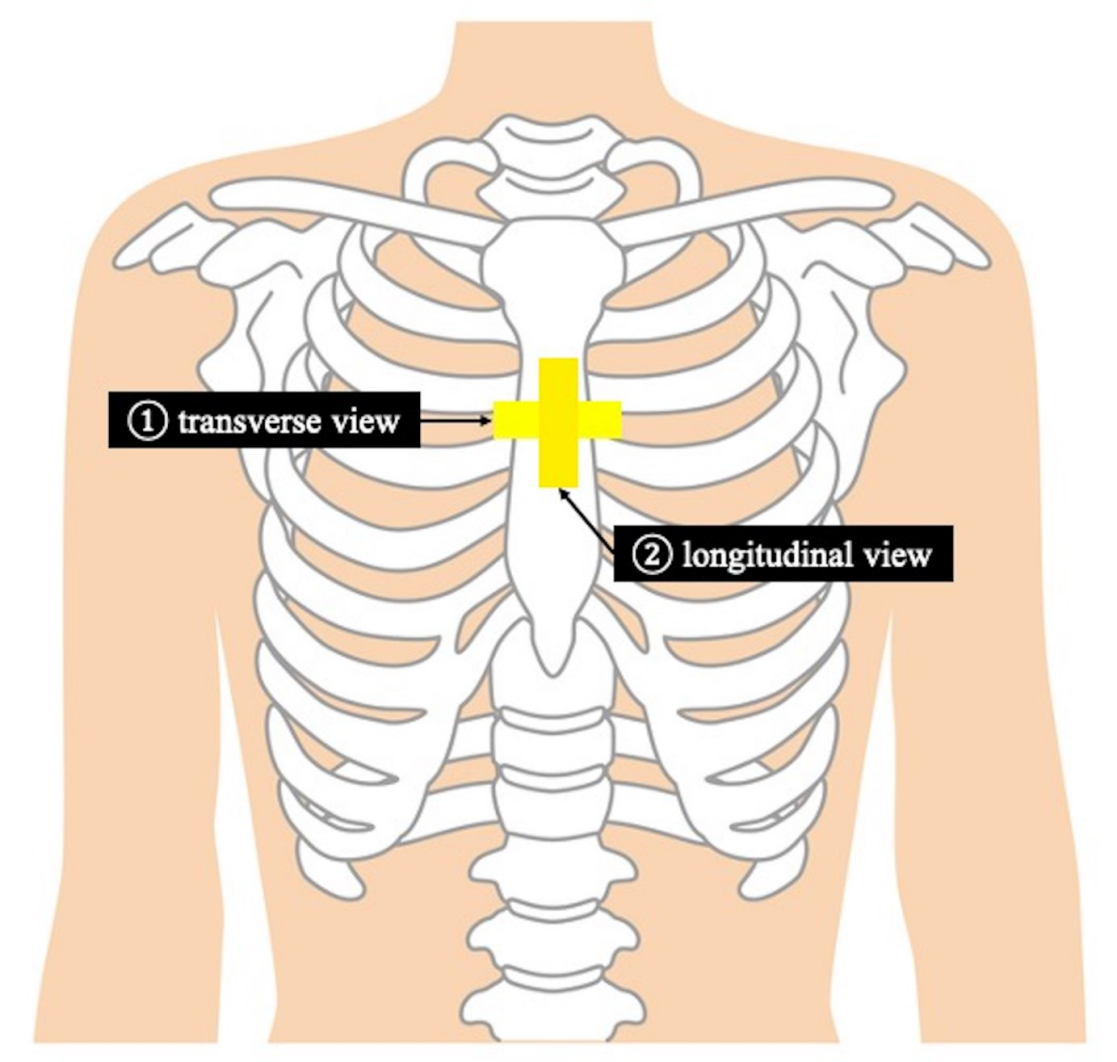
POCUS protocol. A high-frequency linear transducer (8-13 MHz) was placed transversely (1) and longitudinally (2) on the sternum.

**Figure 2:**
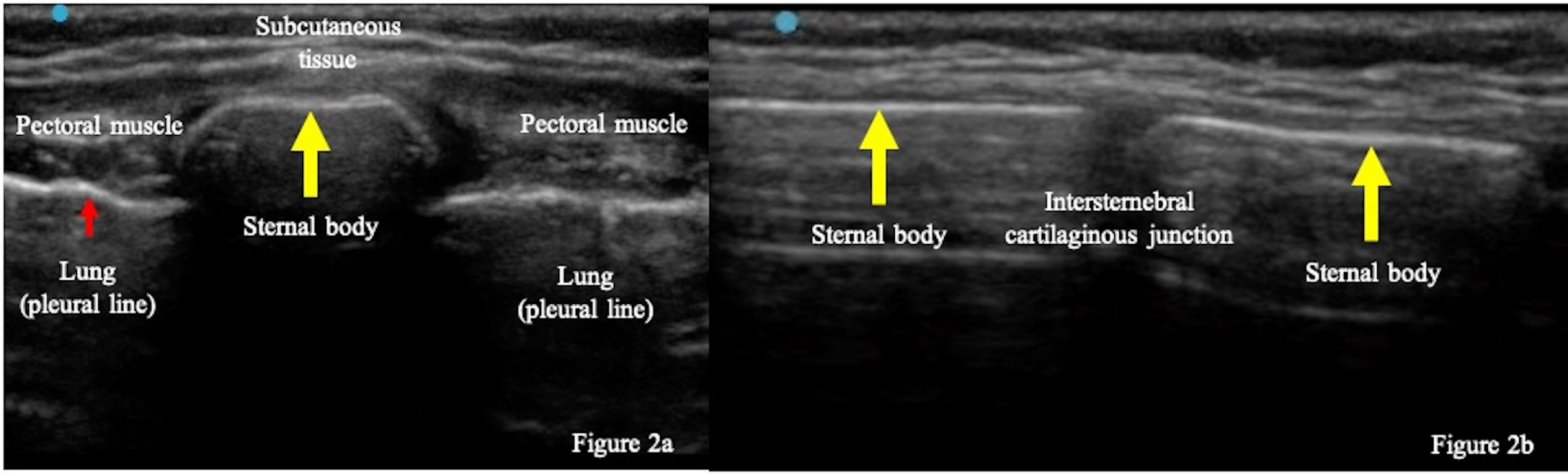
POCUS findings (normal sternum). Transverse (a) and longitudinal view (b) of the sternum showed a hyperechoic structure with acoustic shadows (yellow arrows). POCUS visualized linear cortex of the sternum without discontinuation. (red arrows show the pleural line of the normal lung.)

**Figure 3:**
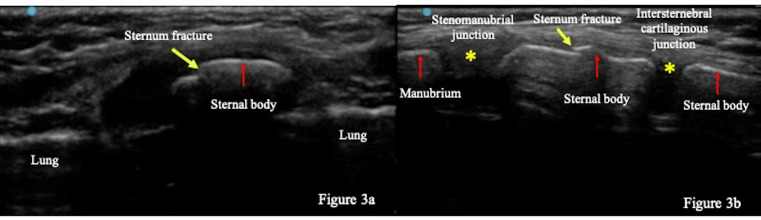
POCUS findings (sternum fracture). Transverse (a) and longitudinal view (b) of the sternum showed a discontinuation of a hyperechoic structure (yellow arrow), suggesting the sternal fracture. (red arrows show the manubrium and sternebra, and asterisks shows the stenomanubrial junction, and intersternebral cartilaginous junction, respectively)

A pediatric emergency physician with five years’ experience using pediatric POCUS performed a scan using Sonosite EDGE II(FUJIFILM, SonoSite, Inc. Japan) with a high-frequency linear transducer (8-13 MHz). The patient was placed in a supine position, and the transducer was placed transversely and longitudinally over the sternum (Figure 1) 9. POCUS normally shows the sternum as a linear hyperechoic structure with acoustic shadow (Figure 2). In our patient, POCUS revealed a discontinuation in the hyperechoic linear structure on the body of the sternum (Figure 3).

**Figure 4:**
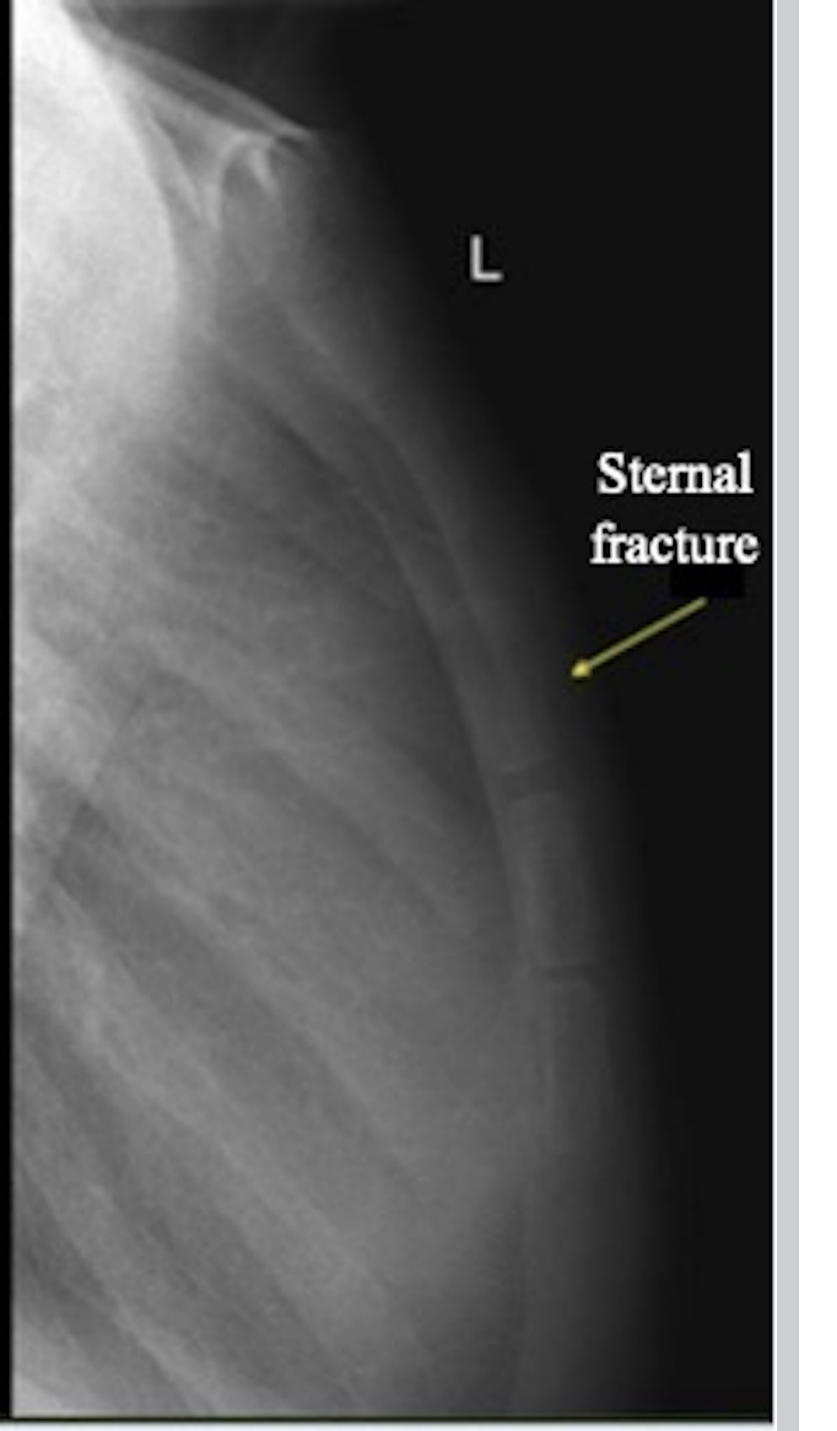
Plain radiograph of the sternum. A discontinuation of the radiopaque structure was detected on the sternal body, suggesting a sternal fracture (arrow).

Based on the findings, the diagnosis of a non-displaced sternal fracture was made. Plain radiography of the sternum confirmed this (Figure 4). Pneumothorax or pulmonary contusion was not reported on the plain radiograph. Electrocardiogram (ECG) showed normal sinus rhythm with no arrythmias or ST-T wave changes. EFAST (extended focused assessment on ultrasonography for trauma) did not reveal pneumothorax or cardiac tamponade, and FoCUS (Focused cardiac ultrasound) showed good left ventricular contraction. The patient was diagnosed with an isolated sternal fracture. He was managed conservatively and discharged from the emergency department. The patient remained asymptomatic and had no complications when reviewed by the cardiac surgeon one week later.

## Discussion

In our case presented, POCUS guided the diagnosis of a pediatric sternal fracture in the emergency department setting. Reports of ultrasound (US) use in diagnosing sternal fractures are scarce. A case-series study demonstrated two cases of pediatric sternum fractures caused by blunt chest trauma, with US performed by radiologists 10. Another case report showed a case of pediatric sternum fracture complicated by subcutaneous abscess. POCUS was performed by a pediatrician six days after the injury due to the swelling of the injury site 11. Only one cases-series study reported POCUS performed by pediatric emergency physician in detecting sternal fractures missed by conventional imaging 12.

Sternal fracture in children is uncommon with its annual prevalence of 0.43/100,000 3. The major mechanism of injury for pediatric sternal fracture are motor vehicle accidents, followed by falls at 67%-77.6%, and 5.9%-6%, respectively 3, 4. However, sternal fractures can occur due to relatively minor trauma such as sports injury or playground fall 13. A retrospective cohort study found that the common concurrent injuries with sternal fracture were extremity fracture (80%), traumatic brain injury (71%), abdominal injury (52%), pulmonary contusion (47%), and rib fracture (38%), with blunt cardiac injury or cardiac tamponade being rare at 0% and 3%, respectively 3. Another retrospective study reported the rates of pneumothorax, rib fractures, and blunt abdominal injuries at 34.8%, 42.8%, and 27.8%, respectively 4. The mortality rate of pediatric sternal fracture was 3.8-8% 3, 4. Moderate or extreme condition in all patients refined diagnosis related group (APR-DRG) category, severe head injury, and mechanical ventilation were independent predictors of mortality, with odds ratios (ORs) and 95% confidence intervals (CI) of 9.80 (7.3-13.16), 16.84 (12.54-22.61), and 3.84 (2.70-5.46). However, isolated sternal fracture was not a risk factor (OR 0.75: 0.34-1.65) for mortality 3. Another retrospective study also stated that isolated sternal fracture can be managed conservatively 5. In our reported case, POCUS not only guided diagnosis of sternal fracture, it was also used to evaluate and rule out concurrent injuries associated with sternal fracture, such as pericardial effusion, intraperitoneal free fluid, and pneumothorax, thus efficiently optimizing safe management.

POCUS has the potential to be an invaluable tool in diagnosing sternal fracture in children. Ultrasonography is more sensitive for diagnosing sternal fractures compared to plain radiography. A meta-analysis of diagnostic accuracy for diagnosing thoracic bone fractures demonstrated that the sensitivity and specificity of US for diagnosing sternal and/or clavicle fractures were 77% (95% CI: 48-100%), and 100% (95% CI: 100-100%), although the patient population was adults and US was performed by radiologists in most of the studies 14. In the pediatric population, no studies investigating diagnostic accuracy of POCUS for diagnosing sternal fracture have been published, but there are a few case reports showing the usefulness of POCUS for this condition 11, 12, 13. A case-series study demonstrated that POCUS can detect sternal fracture despite a normal plain radiography 12. 

POCUS can expedite and enhance management by evaluating for concurrent injuries associated with sternal fracture. As mentioned above, pneumothorax, rib fracture, cardiac tamponade, and abdominal injuries are known associates of sternal fracture, which necessitate rapid intervention 3, 4. The usefulness of POCUS for detecting these conditions has been reported with moderate to high accuracy 14, 15, 16, 17. Therefore, POCUS can aid physicians in optimizing patient management when performed in the appropriate clinical setting.

Last but not least, POCUS is a non-invasive and radiation-free diagnostic modality. CT or MRI are at times required to confirm the diagnosis in patients with suspected sternum fracture despite a reportedly normal radiograph. However, these modalities often require procedural sedation and/or radiation exposure which increases children’s lifetime risk of malignancies 7. POCUS is invaluable as a safe and sensitive alternative tool for diagnosing sternal fracture in children. 

Despite image acquisition and interpretation being operator-dependent and training and experience being required to ensure good quality image 18, pediatric emergency physicians can use POCUS as a highly sensitive and specific modality to diagnose sternal fracture in the same way they use it to diagnose skull and long bone fractures 19, 20. Although further research is needed, this case demonstrates that a pediatric emergency physician can readily use POCUS to diagnose a sternal fracture. 

## Financial support

The present report did not receive any specific grant from any funding agencies in the public, commercial or not-for-profit sectors

## Conflict of Interest

The authors declare that there are no conflicts of interest associated with this manuscript

## Ethical Consideration

Written informed consent to publish details of this case was obtained from the patient’s mother.

## Data availability statement 

Data sharing is not applicable to this article as no datasets were generated or analyzed during the current study.
